# Delivery Systems of mRNA Vaccines in the Treatment of Infectious Diseases: From Lipid Nanoparticles to Next-Generation Platforms

**DOI:** 10.34172/apb.025.46087

**Published:** 2025-10-20

**Authors:** Chou-Yi Hsu, Abdulsalam Abdulsattar Abdulazez, Yasir Qasim Almajidi, A. K. Kareem, Abdullah A. Aseeri, KDV Prasad, Zahraa Khudhair Al-Khafaji, Zuhair I. Al-Mashhadani, Sami Najaf Bokhoor, Raad N Hasan

**Affiliations:** ^1^Department of Pharmacy, Chia Nan University of Pharmacy and Science, Tainan 71710, Taiwan; ^2^Medical Laboratory Techniques Department, College of Health and Medical Technology, University of Al-maarif, Anbar, Iraq; ^3^Department of Pharmaceutics, College of Pharmacy, Alnahrain University, Baghdad, Iraq; ^4^Biomedical Engineering Department, College of Engineering, Al-Mustaqbal University, Hillah 51001, Babil, Iraq; ^5^Department of Clinical Laboratory Sciences, College of Applied Medical Sciences, King Khalid University, Abha, Saudi Arabia; ^6^Symbiosis Institute of Business Management, Hyderabad, Symbiosis International (Deemed University), Pune, India; ^7^College of Pharmacy, the Islamic University, Najaf, Iraq; ^8^Department of Medical Laboratories Technology, AL-Nisour University College, Baghdad, Iraq; ^9^College of Health and Medical Technologies, National University of Science and Technology, Dhi Qar, Iraq; ^10^Biotechnology and Environmental Centre, University of Fallujah, Iraq

**Keywords:** mRNA vaccine, Infectious disease, Lipid nanoparticle, Delivery system

## Abstract

The historic accomplishment of mRNA vaccines against SARS-CoV-2 has provided a massive shift in vaccinology, providing a quick, nimble, and powerful platform for infectious disease prevention. This success, however, does not simply stem from the mRNA sequence but equally depends on the delivery vehicle—the lipid nanoparticle (LNP). The delivery system has evolved from a passive transporter into an active immunomodulatory component, a critical component that (1) protects the inherently fragile mRNA payload, (2) allows cellular uptake and endosomal escape, and (3) adds its own inherent adjuvant properties to shape the immune response. This review provides a comprehensive summary of the current advancements in mRNA vaccine delivery technologies. We first deconstruct the structure, mechanisms, advantages, and disadvantages of the clinically validated LNP platform. Following this discussion, we highlight the emerging landscape of new systems, including chemically diverse polymeric nanoparticles, biologically-inspired peptide-based carriers, and endogenous extracellular vesicles, potentially overcome current limitations in these delivery systems, including issues with thermostability and targeted delivery. After this, we summarize how these new delivery technologies are being leveraged clinically for a continuum of high-priority infectious diseases, including influenza, RSV, CMV, HIV, Zika, and Rabies. This discussion also illustrates how the design of vaccine prototypes is being rational to address the immune-mediated strategies exploited by each distinct pathogen.

## Introduction

 The onset of the COVID-19 pandemic caused by the severe acute respiratory syndrome coronavirus 2 (SARS-CoV-2) was a crisis event in the fields of medicine and vaccinology. The rapid generation, approval, and deployment of vaccines that are highly effective on a global scale is an incredible scientific achievement that changed the landscape of how scientists and public health entities prepare for pandemics.^1^ The success of messenger RNA (mRNA) technology underpinned this pandemic preparedness revolution. Once a promising but marginalized area of research, companies developing mRNA-based platforms entered the global spotlight as they showed profound potential to rapidly create highly potent immunogens to combat newly emerged pathogens.^2^ The use of mRNA in the BNT162b2 (Pfizer-BioNTech) and mRNA-1273 (Moderna) vaccines not only provided critical vaccines to curb the pandemic, but simultaneously showed that mRNA could be a highly valuable, flexible, and powerful tool for therapeutic delivery, marking the dawn of a new era in the prevention and treatment of infectious diseases.^3^ mRNA vaccines, in contrast to traditional vaccine technologies such as live-attenuated or inactivated pathogen technologies, viral vectors, or recombinant proteins, are manufactured using a cell-free synthetic approach. This approach permits very rapid design, scale-up, and adaptations. A new vaccine candidate can be designed and synthesized within days of obtaining the genetic sequence of the target antigen, which was previously unheard of.^4,5^ In addition, mRNA is a non-infectious, non-integrating platform and does not have the risk of insertional mutagenesis, providing an excellent safety profile because they are, in essence, pharmacologically inert. After being delivered into the cytoplasm of the host cell, the mRNA molecule is translated by the ribosomal machinery of the host cell to produce the target antigen in situ. Thus, the intracellular production of antigen completely mimics a natural viral infection in that it will generate robust presentation on MHC class I and class II molecules.^6,7^ Therefore, mRNA vaccines are uniquely positioned to generate a balanced and potent immune response with both a strong neutralizing antibody (humoral) and robust, long-lasting CD4^+^ and CD8^+^T-cell (cellular) immunity necessary for eradicating established infections and long-term prolonged immune protection.^8^

 Nonetheless, the incredible therapeutic potential of mRNA is fundamentally linked to a very challenging issue, the delivery of mRNA. Naked mRNA is an extremely fragile and immunogenic molecule that cannot be administered directly. It is a large negatively charged polyanion that cannot passively diffuse across the anionic lipid bilayer of cell membranes. Naked mRNA will also be quickly degraded by the untold number of ribonucleases (RNases) present in the extracellular space.^9,10^ If naked mRNA were to enter the circulation, it would be rapidly eliminated and could induce a significant systemic inflammatory response by activating pattern recognition receptors (PRRs) such as the Toll-like receptor (TLR) 7 or 8.^11^ Thus, the success of any mRNA vaccine is not simply a function of the message encoded in the mRNA, but critically depends on how well the delivery advances the vehicle’s purpose. This review will provide a high-level update on the newest advancements in delivery systems for mRNA vaccines against infectious diseases and a critical overview of the cognitive constructs, therapeutic action, and clinical efficacy of the current gold-standard delivery methods. We will then provide a glimpse into the frontline of innovation, describing new and emerging materials and next-generation methods designed to enhance targeted tissue delivery, improve thermal stability, and enable innovative administration routes. Lastly, we will discuss unmet needs and future outlooks in the space, in particular bi-directional interactions between the delivery vehicle and the immune system and the regulatory and manufacturing considerations that underscore the future direction of mRNA delivery and vaccines.

## Delivery systems of mRNA vaccines: Structures and mechanism of action

**Figure 1:**
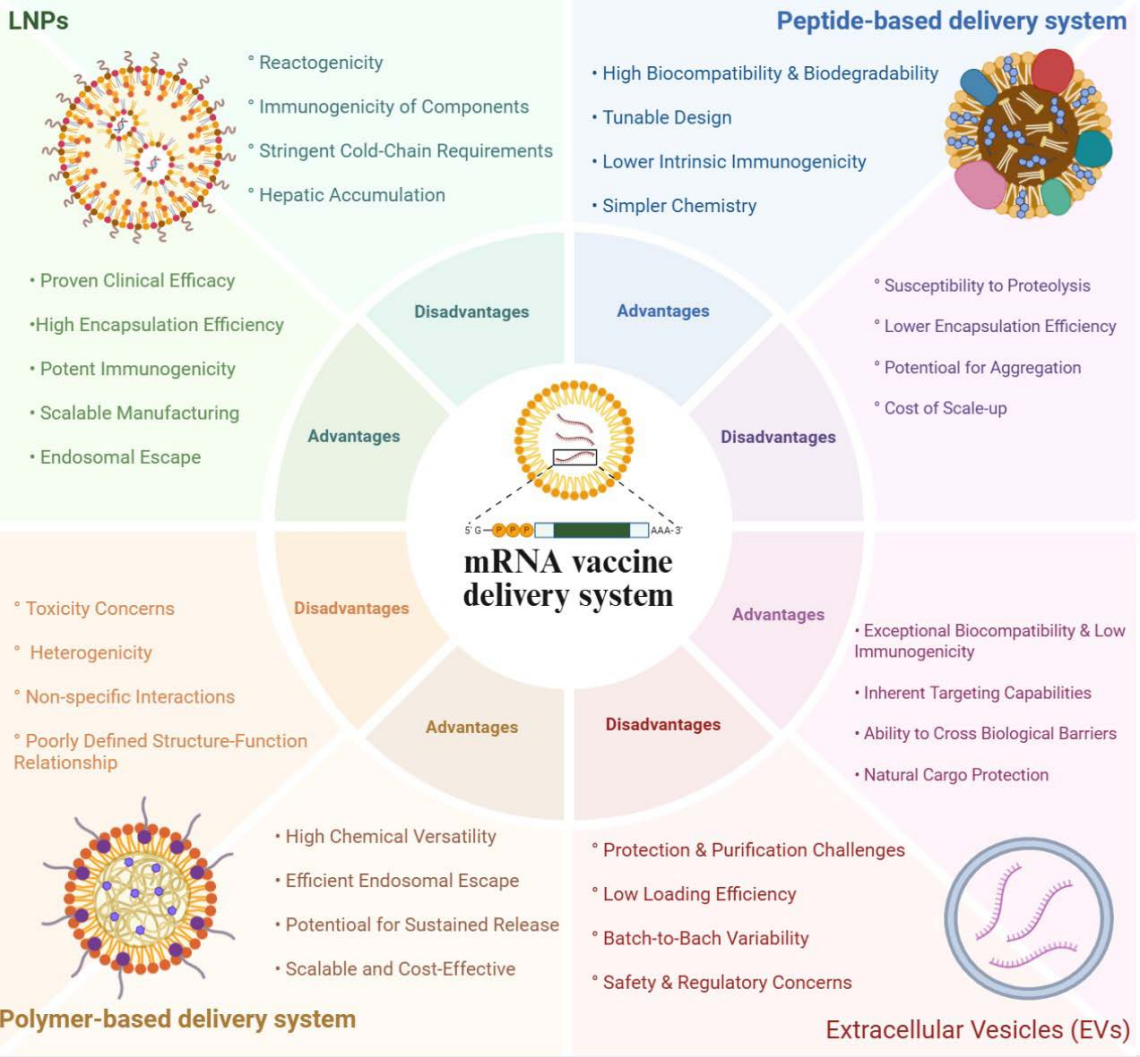
Different types of delivery systems in mRNA vaccines against infectious diseases

 The clinical utilization of mRNA’s therapeutic potential is wholly reliant on its delivery vehicle. An ideally suited delivery system must protect the fragile mRNA from degradation, allow its entry into target cells, liberate it in the cytoplasm, and, preferably, have adjuvant-like properties to augment the immune response. This section addresses some of the major classes of non-viral delivery systems being explored for mRNA vaccines. Figure 1 summarizes different delivery systems and their advantages and disadvantages.

###  Lipid-based systems 

 Lipid nanoparticles (LNPs) are, as of today, the most successful and clinically advanced mRNA delivery platform, and the foundation of the approved COVID-19 vaccines. LNPs are multi-component nanoparticles (typically 80-100 nm) with a solid, electron-dense core instead of an aqueous center. There are four key lipid components in LNP: 1) Ionizable cationic lipid: This is the key. It is positively charged at low pH (during formulation) to bind to negatively charged mRNA and near neutral at physiological pH to reduce toxicity and non-specific *in vivo* binding. 2) Helper phospholipid (e.g., DOPE, DSPC): This is a zwitterionic lipid that forms the nanoparticle structure and assists with stability and membrane fusion. 3) Cholesterol: This is a structural “glue” that fills the spaces between the other lipids, improving the stability of the nanoparticles and performing a regulatory role over membrane fluidity. 4) PEGylated Lipid: This is a lipid covalently linked to polyethylene glycol (PEG). It forms several hydrophilic coronas on the surface of the LNP, providing the steric hindrance that prevents protein opsonization, reduces clearance from the reticuloendothelial system (RES) and ultimately improves circulation time.^12,13^ In the LNP-based systems, lipids and mRNA are mixed in an acidic aqueous buffer (pH ~4). The ionizable lipid is protonated (positively charged) and electrostatically complexes with the mRNA, sequestering it in the resulting nanoparticle.^14^ Upon injection and buffering to a physiological pH (~7.4), the ionizable lipid becomes functionally neutral with significant protection from degradation and clearance afforded by the PEG shield.^15^ LNPs also take up apolipoprotein E (ApoE) in the bloodstream, which helps to facilitate receptor-mediated endocytosis, particularly by hepatocytes, but also that of immune cells (for example, antigen-presenting cells).^16^ Once inside the cell, the endosome matures, with acidification occurring. The low pH again protonates the ionizable lipid, making it positively charged. The charged lipid interacts with the anionic lipids of the endosomal membrane, disrupting the membrane environment, which allows for the LNP to escape into the cytoplasm with its mRNA cargo.^17^ In the cytoplasm, the mRNA is made available for the cell’s ribosomes for translation into the target antigen.

 Regarding their advantages, the LNP delivery system has undergone extensive testing with human clinical trials, particularly the Pfizer/BioNTech and Moderna mRNA COVID-19 vaccines, which had about 95% efficacy and solid safety data in a diverse population, and over five billion doses were administered worldwide, saving millions of lives.^18^ LNPs also exhibit high encapsulation efficiency ( > 90% of the mRNA payload).^19^ Additionally, the pH-responsive ionizable lipids within LNPs are designed to utilize endosomal acidification: upon protonation, they actively interact with anionic endosomal lipids to disrupt membranes and achieve effective release of mRNA into the cytosol—a mechanism responsible for the rapid *in vivo* efficacy seen in large-scale studies.^20^ Proven microfluidic mixing techniques, such as staggered herringbone and chaotic-advection micromixers, can produce LNPs with tightly regulated size distributions, low polydispersity, and high batch-to-batch reproducibility. Furthermore, parallel device architectures and antifouling coatings now available for commercial device design enable us to produce LNPs at liter-per-hour rates without compromising product quality.^21^ However, inherent liver tropism, inflammatory side effects, requiring stringent ultra-cold chain storage (-20 °C to -80 °C), and the intellectual property of ionizable lipids that limit broader research are the main challenges in the development of LNP-based systems for the delivery of mRNA-based vaccines.^22,23^

###  Polymer-based systems

 Polymers provide a highly flexible and chemically varied alternative to lipids for complexing and delivering mRNA. From a structural point, amphiphilic polymers (e.g., poly(ethylene imine) [PEI], chitosan, and poly(β-amino esters) [PBAEs] possess a cationic character that allows for self-assembly with anionic mRNA. The nanoparticles produced through this process are called polyplexes.^24^ The polyplexes’ charge and size can be changed by modulating the polymer-to-mRNA ratio.

 A defining benefit of polymeric vectors is their outstanding suite of chemical diversity. Sophisticated polymer chemistry is able to produce enormous libraries with completely tunable characteristics (charge density, hydrophobicity, molecular weight, degradability, and architecture (linear, branched, or dendritic)).^24,25^ The tunability of the delivery vehicle, along with its ability for rational design, enables optimization of polymers for specific purposes - including delivering siRNA, DNA vaccination, etc. One prominent example is how PBAEs have been formed in libraries through combinatorial synthesis,^26^ which enables streamlined identification of structures with higher transfection efficiency and lower toxicity. This level of structural control becomes a requirement for their high payload capacity. The abundant cationic charge on the polymer backbone can interact strongly with the negatively charged phosphate backbone of the nucleic acids, creating large complexes in the form of polyplexes, while also allowing the protection of the nucleic acid, preventing enzymatic degradation and permitting the unsusceptible formation of large payloads.^27^ Polymeric vectors, in contrast to viral vectors that require complex biomanufacturing processes for synthesis, offer a significantly lower cost and reduced time requirements.^28^ There are several key challenges which stand in the way of successfully clinically translating polyplexes. The first is cytotoxicity, which is an issue that is commonly associated with cationic polymers with high molecular weight that do not degrade. Polyethylenimine (PEI) has long been regarded as a “gold standard” for transfection not just because of its high transfection efficiencies, but also because of its association with potentially toxic effects. For example, PEI’s high density of cationic charge can lead to acutely toxic outcomes when it causes cell membranes to lose their integrity, ultimately resulting in cell death by either apoptosis or necrosis.^29^ A second challenge is the polydispersity of polyplexes themselves. The process of self-assembly results in nanoparticle sizes, charges, and shapes that are not homogeneous, and because the fate of the nanoparticle is so reliant on its physicochemical properties, heterogeneity can lead to batch variation and potentially unpredictable *in vivo* behavior. This could cause problems for quality control and regulatory approval processes, and could lead to differing therapeutic efficacy.^30^ Lastly, polyplexes also tend to demonstrate poor serum stability. Once entering the blood circulation, many positively charged polyplexes will non-specifically interact with anionic serum proteins, coating the polyplex, and forming a “protein corona.” This opsonization leads to very rapid aggregation and clearance of the polyplexes from circulation by the RES in the liver and spleen, thus significantly reducing the chances of reaching the target tissues.^31,32^

###  Peptide-based systems

 Peptides represent a biologically inspired approach by utilizing precise sequences of amino acids to perturb cellular barriers. Structurally, they are short chains of amino acids (generally 5-30), that are typically cationic and/or amphipathic. They associate with mRNA to form nanoscale complexes through electrostatic and hydrophobic interactions. Notable examples include cell-penetrating peptides (CPPs) such as TAT peptide or synthetic cationic amphipathic peptides that are designed to perturb membranes.^33,34^ In this system, CPPs can mediate uptake through different endocytic pathways, or potentially direct translocation in high-concentration conditions where mRNA would simply cross the plasma membrane. Amphipathic peptides are designed to change conformation in an acidic environment, like the endosome. Their hydrophobic domains perturb the endosomal membrane through two mechanisms: either insertion into the membrane, creating a pore or destabilization of the bilayer to release cargo.^34^ Although peptide-based systems have several advantages, including biodegradability, high cell penetration, and targeting potential,^35^ some challenges limit their effectiveness, such as rapid degradation by proteases in the bloodstream (poor *in vivo* stability), immunogenicity, and the strong binding between peptides and mRNA, which can hinder the release of mRNA and diminish the levels of target protein expression.^33,34^ It is worth noting that peptides are often used as part of hybrid systems rather than as standalone carriers.

###  Biologically-derived systems (Extracellular vesicles)

 Extracellular vesicles (EVs) are an exciting class of natural nanocarriers for therapeutic biomolecules, in particular, for RNA-based medicines. Due to their innate biological functions and structural characteristics, EVs offer a unique platform that circumvents many of the challenges encountered when developing synthetic delivery systems. They represent a heterogeneous group of cell-secreted vesicles that are encapsulated by a lipid bilayer involved in the process of intercellular communication. This heterogeneous population contains exosomes, which are derived from an endosomal origin and are usually between 30 to 150 nm in size, but also encompasses different-sized microvesicles from the plasma membrane of progenitor cells.^36^ Many consider EVs to be nature’s cargo-couriers, transporting biovariants such as proteins, lipids, and nucleic acids, among them annotated RNA classes of mRNA and miRNA, between cells.^37^ The surfaces of EVs are endowed with a complex arrangement of proteins (e.g., tetraspanin proteins include CD9, CD63, and CD81) and lipids inherited from their parent cell, which then establish their biological identity and interaction properties.^38^ As a natural-cargo carrier, nature has improvised on EV cargo delivery and mobilized EVs to account for even further intercellular communication through a myriad of internalization pathways, including both clathrin- and caveolin-mediated endocytosis, macropinocytosis or direct fusion with the plasma membrane. Once internalized, the endogenous protein machinery, and lipid content of the EV membrane are presumed to promote endosomal escape and subsequent efficient cargo delivery.^39,40^

 There are several advantages EVs possess over synthetic nanoparticles that give them promise as therapeutic agents. First, owing to their endogenous nature, the immunogenicity and toxicity of EVs are very low, reducing adverse reactions and enabling a possible repeated therapeutic application.^41^ Second, EVs have a natural tropism for particular cells or tissues. The specificity for targeting a particular cell or tissue is derived from the parent cell and the types of surface proteins (integrins, adhesion molecules, etc.) that are displayed on the EV.^42^ Third, of the many advantages EVs have, their ability to move freely across significant physiological barriers is substantial. In particular, subsets of EV populations have been shown to cross the blood-brain barrier, which poses a challenge for most therapeutics. This has been demonstrated in landmark studies that showed engineered exosomes delivered siRNA across the blood-brain barrier in mice, indicating promise for the treatment of CNS disorders.^43^ Despite their potential, several barriers must be addressed before EV-based therapies reach the clinic. A key bottleneck is developing robust, scalable, and cost-effective clinical-grade EVs, made in accordance with Good Manufacturing Practice (GMP). Current isolation methods, such as ultracentrifugation, size-exclusion chromatography (SEC), and immunocapture, demonstrate a compromise between yield and purity. Separating EVs from biological contaminants, such as lipoproteins and protein aggregates, is a very difficult technical challenge.^44,45^ While EVs carry RNA by nature, loading EVs with high concentrations of exogenous therapeutic mRNA is a major technical challenge. Methods like electroporation, sonication and transfecting producer cells are currently being evaluated to load EVs, all of which can damage the vesicle membrane, degrade the RNA cargo, or lead to low and inconsistent loading efficiency, which complicates the development and dosing of therapeutics.^46^

###  Comparative analysis and translational challenges of delivery systems

 Although LNPs have established a gold standard for the delivery of mRNA-based vaccines, it is apparent that the advancing space of polymer-based, peptide-based, and EV-based systems offers distinct advantages and demonstrates solutions to some of the intrinsic disadvantages of LNPs. For instance, LNPs are explicitly intended for non-mucosal systemic intramuscular (IM) injection in the body,^47^ and therefore, it is questionable as to whether they will penetrate mucosal barriers and induce the level of local immunity (e.g. IgA responses) required for respiratory or gastrointestinal pathogens. In the area of mucosal delivery, several polymeric systems, particularly ones in which mucoadhesive materials like chitosan are utilized, indicate possibilities of providing some benefits over LNPs.^48^ Polymeric nanoparticles also can be specifically engineered to achieve targeted mucoadhesion and deliver for controlled release for desired immunogenicity, and may support intranasal or oral delivery mechanisms.^49^ Thermostability also provides another important distinction among delivery systems. Whereas LNPs require ultra-cold (-20 °C to -80 °C) storage chains,^50^ cold chain storage and shipping of vaccines to global destinations poses serious logistics issues, especially in low-resource or low-tech settings. While advances in LNP formulations are underway, polymer-based systems are more easily designed to remain stable (or stabilized) at higher temperatures, including through lyophilization, and through polymers that inherently afford better protective elements for wild-type mRNAs from environmental factors.^51-53^

 Regarding safety and immunogenicity, all the delivery systems have unique profiles. LNPs are considered to be clinically proven, but have been shown to cause inflammatory side effects, and the ionizable lipids can contribute to reactogenicity.^54,55^ Polymeric systems, primarily those with a higher charge density, can be cytotoxic and therefore require designing degradable polymers very carefully.^56^ Peptide systems can often superficially appear biodegradable but can create immunogenicity against the peptide itself. Moreover, due to instability and degradation from proteases *in vivo*, there is an *in vivo* limitation.^57^ EVs are of natural origin and therefore often have low immunogenicity and toxicity, providing an advantageous safety profile for repeated doses. However, the potential immunomodulatory effects derive from their cellular origin and the risk for unintended off-target effects will need to be balanced and should be examined.^58^ Targeted delivery is a significant innovation area. LNPs do have some tropism (e.g., liver tropism via ApoE uptake), but there is still a need for increased cell or tissue-specific targeting.^59^ Polymeric nanoparticles have high chemical tunability that allows for surface functionalization with targeting ligands (e.g., antibodies, peptides), enabling possible improvements in specificity.^60^ Similarly, although EVs have specific targeting capabilities on the surface of their proteins, such proteins can potentially be engineered for tropism to specific cells, meaning that there may be ways to circumvent any limited off-target biodistribution effects associated with EVs.^61^ Additionally, peptide-based systems mainly involving CPPs (which are inherently designed to permeate cells) can be modified for enhanced targeting specifically.^62^

 Manufacturing and scalability are considered essential barriers in the translational of all advanced delivery systems. Although LNP production can be scaled to billions of doses, some of the useful ionizable lipid’s proprietary nature may limit further research and development of the LNP systems.^63^ Whereas polymeric systems present much flexibility at the raw material levels and also at the synthesis level, polydispersity of polymeric systems can greatly complicate quality assurance, as well as make regulatory approval more difficult.^64^ Moreover, there is evidence that PEGylation of polymeric nanoparticles could exhibit non-biodegradability in clinical applications, which adversely affects their renal excretion and increases hepatotoxicity. PEGylated polymeric nanoparticles also showed hypersensitivity reactions and eliciting the production of antibodies against PEG.^65^ EVs currently are at the greatest disadvantage for manufacturing due to robust and scalable GMP manufacturing considerations, and are there advantages will include efficient removal of biological contaminants, and the ability to achieve a high and consistent levels of loading of exogenous mRNA into EVs, thus providing for cost-effective clinical translation.^66,67^

## mRNA vaccine for infectious diseases

**Figure 2:**
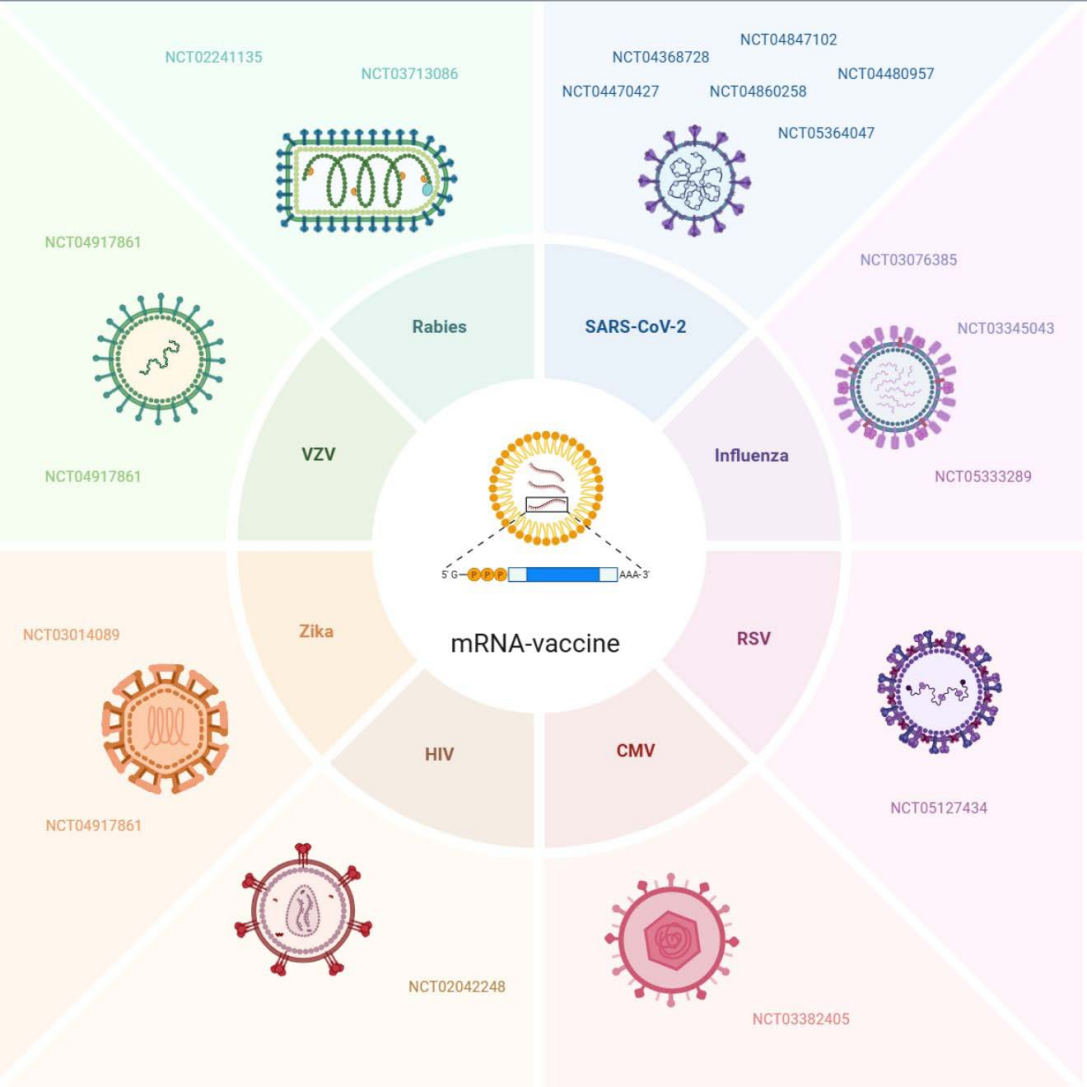
Clinical trial of mRNA-based vaccines for infectious diseases, such as SARS-CoV-2, influenza,RSV, CMV, HIV, Zika, VZV, and rabies

 Researchers have developed mRNA vaccines that target specific antigens to hopefully mitigate infectious diseases that are being tested in clinical trials. mRNA delivery as a method of prevention or treatment is a viable option created by researchers, as it is effective and has fewer side effects than previous methods. In this case, it was established as an effective way to obtain immunoprotection against COVID-19. So far, research has mainly focused on mRNA vaccines in regard to preventing or treating infectious diseases such as SARS-CoV-2, influenza, respiratory syncytial virus (RSV), cytomegalovirus (CMV), human immunodeficiency virus (HIV), varicella-zoster virus (VZV), Zika virus, and rabies, as illustrated in Table 1 and Figure 2.

**Table 1:** Clinical trials of mRNA vaccines for infectious diseases

**Pathogen**	**Vaccine**	**Antigen**	**Phase**	**Delivery system**	**Status**	**NCT number**
SARS-COV-2	mRNA-1273	Full-length spike	III	LNP	Completed	NCT04470427
	BNT162b1, BNT162b2	Spike (RBD)	II/III	LNP	Completed	NCT04368728
	LUNAR-COV19	Spike (2 proline)	I/II	LNP	Completed	NCT04480957
	CVnCoV	Spike (2 proline)	III	LNP	Terminated	NCT04860258
	ARCoV	Spike (RBD)	III	LNP	Recruiting	NCT04847102
	LNRNA009	Spike (RBD)	I	LNP	Recruiting	NCT05364047
	NVX-CoV2373	Full-length spike	II/III	Protein nanoparticles	Completed	NCT05925127
Influenza	VAL-506440	HA	I	LNP	Completed	NCT03076385
	VAL-339851	HA	I	LNP	Completed	NCT03345043
	mRNA-1010, mRNA-1020, mRNA-1030	HA, NA	I/II	LNP	Completed	NCT05333289
RSV	mRNA-1345	F (fusion) glycoprotein	II/III	LNP	Active, not recruiting	NCT05127434
	JCXH-108	F (fusion) glycoprotein	I	LNP	Active, not recruiting	NCT06564194
CMV	mRNA-1443, mRNA-1647	gB, pentamer complex, pp65	I	LNP	Completed	NCT03382405
	mRNA-1647	gB, pentamer complex	III	LNP	Active, not recruiting	NCT05085366
HIV	AGS-004	HIV-1 antigens	I	DC	Completed	NCT02042248
	mRNA-1644, mRNA-1644v2-Core	eOD-GT8 60-mer, Core-g28v2 60-mer	I	LNP	Active, not recruiting	NCT05001373
Zika	mRNA-1893	prM-E	II	LNP	Completed	NCT04917861
	mRNA-1325	prM-E	I	LNP	Completed	NCT03014089
Rabies	CV7202	RABV-G	I	LNP	Completed	NCT03713086

LNP, lipid nanoparticle; HA, hemagglutinin; NA, neuraminidase; RSV, respiratory syncytial virus; CMV, cytomegalovirus; gB, glycoprotein B; pp65, phosphoprotein 65; DC, dendritic cell; prM-E, pre-membrane (prM) and envelope (E); RABV-G, rabies virus glycoprotein G.

###  mRNA vaccines for SARS-CoV-2

 The COVID-19 pandemic, linked to the SARS-CoV-2, represented a global health crisis of the highest magnitude. It was also an important proving ground for mRNA vaccine technology, advancing from a “promising” development phase to a platform of modern vaccinology.^68^ The remarkable speed of developing, authorizing, and rapidly deploying both bNT162b2 and mRNA-1273 is particularly notable because they effectively rely on complex and excellent delivery systems. This part will evaluate the delivery systems of both first-in-class SARS-CoV-2 vaccines, outlining their components, mode of action, and the factors that influenced their design. The primary antigenic target for SARS-CoV-2 vaccines is the viral spike (S) glycoprotein. The S protein is a large, trimeric transmembrane protein located on the virion surface that facilitates host cell entry by binding the angiotensin-converting enzyme 2 (ACE2) receptor.^69^ Neutralizing antibodies that obstruct this interaction are a major correlate of protection. Thus, the mRNA vaccines were developed to contain a stabilized (prefusion) conformation of the S protein. To stabilize, mRNA sequences included two proline substitutions (K986P and V987P), collectively termed the “2P” mutation. The “2P” mutation holds the S protein in its prefusion conformation, more effectively exposing critical neutralizing epitopes and producing a more potent and focused antibody response.^70,71^ In addition to the antigen-coding sequence, the mRNA molecule itself was extensively modified to enhance protein expression and reduce innate immune activation, which could otherwise lead to degradation. A major advance was substituting the uridine of mRNA for N1-methylpseudouridine (m1Ψ). This nucleoside modification reduces the activation of the local innate immune sensors, such as TLR7/8 and RIG-I, in a way that prevents immediate clearance of the mRNA, allowing for greater translational efficiency and duration. Finally, the mRNA construct is also surrounded by 5’ and 3’ untranslated regions (UTRs) and a poly(A) tail, both of which contribute to the stability of the molecule as well as ribosome recruitment.^72,73^ It is worth noting that the ionizable cationic lipid in the structure of LNPs is the most important part and the heart of the proprietary technology. At an acidic pH (e.g., when formulating, pH ~4), these lipids are positively charged, in which case they can complex with the negatively charged mRNA backbone via electrophilic interactions. With a physiological pH (~7.4), they become near-neutral. The “smart” pH-responsiveness is important. The neutral charge in circulation helps avoid non-specific binding to the components of blood occluding and any potential toxicities. Upon uptake into a cell via endocytosis, the endosome will acidify (pH ~5.5-6.5) and reprotonate the lipid charge to positive. The reprotonation of the lipids is critical to disrupt the endosomal membrane and allow the release of the mRNA cargo into the cytoplasm– the key “endosomal escape” step. The ALC-3015 in the Pfizer-BioNtech vaccine and the SM-102 in the Moderna vaccine act as ionizable cationic lipids.^74^

 The Moderna vaccine is formulated with the use of LNP along with the ionizable lipid SM-102 to deliver the modified mRNA. This modified mRNA carries N1-methylpseudouridine and encodes the spike protein of SARS-CoV-2 with 2 proline changes (S-2P). Strong immune responses against both the original virus and the D614G variant of concern were observed with this vaccine in 2020.^75,76^ After a Phase 1 clinical trial in humans that established safety and efficacy, the mRNA-1273 vaccine was approved in 2020. Individuals receiving the mRNA-1273 vaccine had neutralizing antibody concentrations for the S-2P protein of 299,751, 782,719, and 1,192,154 after their second shot with 25 µg, 100 µg, or 250 µg doses, respectively. Although the studies revealed increased immunogenicity with the higher doses of mRNA-1273, they also revealed an inflammatory reaction from T-cells, resulting in complications for some vaccines. Of the 250 µg dose recipients, approximately 21% experienced severe adverse effects.^77^ Later that year, mRNA-1273 proceeded to a Phase II/III trial in children aged 6-11 years. In the first portion of the trial, there were 751 children that received the 50 µg and 100 µg doses of the vaccine, with the lower dose moving forward because it showed greater promise. In the second portion of the study, 4016 children were randomly assigned to either 50 µg of vaccine or a placebo 82 days after the first dose. Side effects in the children were generally mild including minimal pain at the injection site, fatigue, and headaches. A month after the second dose, neutralizing antibody levels of 1610 had been achieved in the children receiving the 50 µg and 1300 in adults receiving the 100 µg dose. Overall, mRNA-1273 had an approximate efficacy of 88% in preventing infection, which was rather promising.^78^ Pfizer and BioNTech developed two mRNA vaccines called BNT162b1 and BNT162b2, with the structure of mRNA altered to improve translation. The BNT162b1 vaccine uses the S glycoprotein (RBD) and the BNT162b2 vaccine uses the S-2P protein. The first clinical trial of these vaccines occurred in 2020 when healthy volunteers received one of the two vaccines as an initial dose. After 21 days, the second dose, developed from a vaccine or a placebo dose, was randomly assigned to volunteers. Volunteers who were vaccinated received different doses of the vaccines: 10 µg, 20 µg, 30 µg, and 100 µg. Both vaccines produced an immune response to COVID-19, with the peak neutralizing immune response recorded 14 days after the second shot.^79,80^ BNT162b1 and b2 vaccines quickly elicited strong immune responses and a side effects assessment revealed that BNT162b2 (also shown in older adults) generated less severe systemic side effects than BNT162b1.^80^ The BNT162b2 vaccine induced a strong follicular T helper cell response in humans.^81^ In 2022, a follow-up study involving 51 people assessed the ability of the BNT162b2 vaccine to provide immunity against the beta, delta, and Omicron variants with two or three doses. They found the second dose resulted in a 22-fold increase in neutralizing antibody levels against Omicron and increased neutralizing levels by 23-fold after the third dose.^82^

 ARCoV, another SARS-CoV-2 vaccine, was also developed utilizing mRNA, encoded for the RBD gene, with its first employment with LNP technology in 2020. Testing in mice showed that ARCoV can elicit productive neutralizing antibodies as well as robust cell-mediated immunity against the virus. Mice that received the ARCoV vaccine were protected from SARS-CoV-2.^83^ Macaques (a nonhuman primate model), when administered two doses of ARCoV, developed robust immune responses, both humoral and cellular, to stimulate immunity against SARS-CoV-2. Preclinical evidence in macaques indicated complete protection of the lungs from SARS-CoV-2 infection by ARCoV.^84^ It is important to note that ARCoV began Phase III clinical trials in Mexico and Indonesia (NCT04847102) in 2021, but more information is needed regarding its efficacy and any adverse effects. In 2020, another mRNA vaccine, LUNAR-COV19 with the S-2P gene, was studied.^85^ The immune response was evaluated with a human ACE2 transgenic C57BL/6 mouse model and included safety evaluations that showed LUNAR-COV19 generated an immune response increasing neutralizing antibody levels, and based on these favorable immune outcomes, LUNAR-COV19 entered a Phase II clinical trial in humans (NCT04480957), which also raised neutralizing antibodies among participants and required additional safety data to understand the immune response in more detail. Table 2 summarizes mRNA-based vaccines that have employed delivery methods other than LNPs against COVID-19.

**Table 2:** mRNA-based vaccines employed delivery methods other than LNPs against COVID-19

**Delivery system**	**Antigen**	**Administration route (Dosage)**	**Model**	**Outcome**	**Ref**
Exosome	Spike, LSNME	IM (0.25 and 4 μg)	Mice	↑Antibody and T responses, No adverse reactions	^86^
CARTs	Spike (RBD)	IV (3 μg)	Mice	↑Antibody and T cell memory, No liver toxicity	^87^
Exosome	Spike (RBD)	ID (0.5 mg)	Mice	↑Antibody responses	^88^
Lipid-polymer hybrid	Full-length spike	IM (10 μg)	Mice	↑Antibody and Th1 responses	^89^
PGS conjugate	Spike (RBD)	IM (10 μg)	Mice	↑Antibody responses	^90^
oEV	Spike S1 subunit	IM (60 μg), IN (60 μg), Gavage (100 μg)	Mice	↑Antibody and Th1 responses, No toxicity	^91^
Chitosan-lipid nanoparticle	Spike (RBD)	IN (2 μg)	Mice	↑Local antibody responses without systemic responses	^48^
PNP hydrogel	Full-length spike	SC (50 μg)	Mice	↑APC recruitment, ↑Antibody responses	^92^
Liposome	Spike (RBD)	IV, IM, SC, ID, and IP	Mice	↑Antibody and Th1 responses	^93^
Liposome	Full-length spike	IM (50 µL)	Mice	↑Humoral and cellular responses	^94^

LSNME, nucleocapsid protein and fragments of the spike, membrane, and envelope; IM, intramuscular; CARTs, charge-altering releasable transporters; IV, intravenous; ID, intraduodenal; PGS, polyglucin: spermidine; oEV, orange-derived plant extracellular vesicle; IN, intranasal; PNP, polymer-nanoparticle; SC, subcutaneous; APC, antigen-presenting cell, ID, intradermal; IP, intraperitoneal

###  mRNA vaccines for the influenza virus

 The potential promise of mRNA technology for influenza is currently undergoing rigorous assessment in preclinical models and human clinical trials. The delivery system represents a critical determinant of a vaccine’s overall success, influencing the stability, immunogenicity, safety, and scalability of the end product. The unprecedented success of the SARS-CoV-2 mRNA vaccines has established LNPs as the new gold standard for delivering mRNA. LNPs are a multi-component system designed to protect the mRNA payload from degradation, deliver the payload to the cell, and promote endosomal escape from the endosomic compartment of the cell. Leading vaccine developers (e.g., Moderna and Pfizer/BioNTech) have utilized their validated LNP platforms to develop influenza vaccine candidates, which have advanced to late-stage clinical trials. Moderna has created a multi-faceted influenza vaccine program using its unique LNP formulation, the same one utilized in the Spikevax COVID-19 vaccine. Their plan includes bridging seasonal quadrivalent, pandemic-ready, and combination respiratory vaccines. The mRNA-1010 (seasonal quadrivalent vaccine) encodes the hemagglutinin (HA) glycoproteins of four seasonal influenza strains (H1N1, H3N2, B/Victoria, and B/Yamagata), recommended by the WHO.^95^ The vaccine was evaluated in a Phase 1/2 study in younger and older adult cohorts. The vaccine used was generally well-tolerated with an adverse event profile similar to previous mRNA vaccines (e.g., injection site pain, fatigue, myalgia). Immunologically, mRNA-1010 stimulated strong antibody responses, with geometric mean titers (GMTs) against each of the four strains increasing post-vaccination in a statistically significant manner. In several arms of the study, these GMTs were equal to or higher than those for approved quadrivalent influenza vaccines.^96,97^ In contrast, the subsequent Phase 3 study (P301) showed more subtle results. While the immunogenicity of mRNA-1010 was determined to be non-inferior against the A strains (H1N1, H3N2) of a licensed standard-dose vaccine, it was not non-inferior for the two B strains (Victoria and Yamagata).^98^ This result brings us to a critical issue: balancing the potency in the pandemic response against all four strains, especially the less immunogenic B lineages, using an LNP-mRNA platform. Moderna is moving forward on next-generation candidates (mRNA-1011 and mRNA-1012) with updated antigens to solve this issue. Moderna also combined mRNA-1010 with mRNA-1273 (Spikevax) in a single LNP formulation, called mRNA-1073. The early-stage trials (Phase 1/2) demonstrated a strong antibody response for both SARS-CoV-2 and all four influenza strains with the combination vaccine. The safety profile was acceptable; however, reactogenicity remains a primary surveillance concern with combination vaccines. The success of this combination product largely rests on the ability of the delivery system to co-deliver multiple mRNA payloads effectively with no antigen interference.^99^ Similar to Moderna, Pfizer and BioNTech are also advancing a quadrivalent seasonal influenza mRNA vaccine candidate, named the BNT161 program, owing to their LNP technology and actively following Phase 3 trials to establish safety and efficacy of their programs.^100^

 In addition to LNP formulations, other delivery systems have also been developed for mRNA-based vaccines against the influenza virus, such as polymer-based and peptide-based delivery systems. For instance, Hardenberg et al clearly illustrated the strong potentials of a poly(amido)amine (PAA)-based nanoparticle system in the delivery of an influenza mRNA vaccine. The authors developed nanoparticles (ps-PAAQ NPs) that contained a modified mRNA encoding for the pandemic A/California/07/2009 (H1N1) virus hemagglutinin (HA). Upon intramuscular administration into ferrets, which are considered a parasympathetic gold standard animal model for human influenza infection due to their similarities in respiratory physiology and disease pathology, the vaccine produced strong and balanced immune responses with only minor local or systemic reactogenicity. Vaccinated animals developed strong humoral immunity, which was characterized by high hemagglutination inhibition (HAI) titers and a robust cellular response, as demonstrated by the significant induction of influenza-specific IFN-γ-secreting T-cells. The final measure of success was the protective ability of the vaccine; following viral challenge vaccinated ferrets still experienced full protection from severe disease, marked by significantly reduced clinical signs and nasal viral loads (compared to challenge control animals).^101^ In another study, Hajam et al transitioned from injectables to a non-invasive protein-coated chitosan nanoparticle system for intranasal delivery. Natural biodegradable polysaccharides that are mucoadhesive, such as chitosan, make great candidates for mucosal delivery of antigens. The researchers encapsulated mRNA molecules coding for 2 highly conserved influenza antigens-the HA stalk domain (HA2) mRNA and the M2 ectodomain (M2e) mRNA-and coated the nanoparticles with the nearly identical proteins with a goal to elicit broad cross-strain protection. The findings of this study, which utilized chickens under an avian influenza (H9N2) challenge, exemplified the power of this hybrid protein-mRNA approach. The intranasal delivery of the chitosan-based vaccine induced both systemic antibody responses (serum IgG) and critical mucosal immunity, as indicated by significant concentrations of IgA in lung washes.^102^

###  mRNA vaccines for RSV

 RSV is responsible for a considerable amount of acute lower respiratory tract infections and a global burden on health care, especially for infants, young children, older adults, and immunocompromised patients.^103^ The primary antigen for neutralizing antibodies is the RSV fusion (F) glycoprotein that exists in two major conformations: a metastable prefusion (pre-F) state and a stable postfusion (post-F) state. Structural biology studies were fundamental in establishing that many of the most potent neutralizing antibodies targeted the pre-F conformational epitopes.^104^ By engineering specific stabilizing mutations (e.g., DS-Cav1), scientists were able to “lock” the F protein in the prefusion conformation, providing a better immunogen.^105^ The mRNA platform is uniquely positioned to deliver the genetic instructions for this stabilized pre-F antigen, thus ensuring that the antigen will be expressed in high-fidelity within the host cell and presented to the immune system in the ideal conformation to elicit a robust neutralizing antibody response. The clinical effectiveness of this LNP-mRNA approach has been established without reservation. Moderna’s vaccine mRNA-1345, which contains an mRNA that encodes for a predetermined pre-F protein embedded in a proprietary LNP, demonstrated vaccine efficacy of 66.7% against RSV-associated lower respiratory tract disease (LRTD) in adults ≥ 60 years of age in its pivotal Phase 3 trial. The immune profile was indicative of a protective, Th1-biased response, directly eliminating the safety concerns of the field that had persisted for over 50 years.^106,107^ Moreover, Pfizer has incorporated a pre-F RSV antigen into its bivalent mRNA vaccine programs against both influenza and COVID-19 and also utilized LNP technology.^108^ Together, these achievements have established LNP-mRNA as the gold standard for systemic RSV mRNA vaccination.

 While LNPs have enabled the first wave of successful RSV mRNA vaccines, the field continues to innovate. For example, Chai et al developed mRNA encoding the stabilized PreF antigen with an ESCRT/ALIX-binding region (EABR) in the cytoplasmic tail. This engineering allows the self-assembly and secretion of enveloped virus-like particles (eVLPs) as transfected host cells generate and export the mRNA, ultimately giving rise to a secondary, particulate mechanism of antigen presentation that approximates a naturally occurring viral entity. In animal models, this PreF-EABR mRNA vaccine was superior to a traditional, unmodified PreF mRNA vaccine, with robust and more durable neutralizing antibody titers that consistently lasted at least 20 weeks afterwards. The clearer and longer-lasting humoral responses correlated with stronger germinal center B cell and T follicular helper (Tfh) cell responses that led to a greater frequency of long-lived memory B cells. Additionally, the PreF-EABR vaccine protected against RSV infection at a lower dose than the traditional PreF mRNA vaccine. Mechanistically, they relate their stronger immunogenicity to the unique transcriptomic profile of the eVLPs. Compared to previously reported Genomic RNA signatures of vaccine-induced antibody longevity, the eVLPs activated the strongest enrichment patterns in the Toll-like receptor signaling pathways and activated platelet-associated signaling pathways.^109^

###  mRNA vaccines for CMV

 Human CMV is still considered a global public health priority and it remains the most common infectious cause of congenital birth defects. Congenital CMV (cCMV) infection causes permanent disabilities such as sensorineural hearing loss, visual impairment, and neurodevelopmental delays. Geared towards the perinatal period, CMV also significantly presents a burden to immunocompromised individuals like solid organ transplant recipients or hematopoietic stem cell transplant recipients, where reactivation can present with life-threatening end-organ disease.^110,111^ A major problem facing CMV vaccinology is how the virus uses different glycoprotein complexes to mediate entry into different cell types. Glycoprotein B (gB), the most abundant surface glycoprotein, is required for all types of entry, but it is the predominant target for neutralization of infection in fibroblasts. However, to prevent viral dissemination and transplacental infection of the fetus, it is necessary to also neutralize the entry into endothelial and epithelial cells. This activity is accomplished via the Pentameric Complex (PC), a five-protein assembly which contains gH, gL, and the UL128, UL130, and UL131A subunits.^112,113^ Neutralizing antibodies targeting the PC are much more effective at blocking epithelial/endothelial cell infection than those targeting gB. CMV utilizes a variety of immune evasion strategies to persist for the lifetime of the host by establishing latent infection, where the virus remains undetectable by host immune surveillance, essentially preventing the development of immunity. Some of the immune evasion strategies used by CMV include preventing antibody-dependent cellular cytotoxicity (ADCC) and/or antibody-dependent cellular phagocytosis (ADCP) using viral Fcγ receptors (vFcγRs) such as gp34 and gp68, regulating natural killer (NK) cells via MHC-I homologs (such as UL18), altering NK cell activating ligands (such as by UL141), downregulating MHC class I molecules, altering CD8^+^T-cell recognition, and producing viral cytokines that prevent DCs from maturing and/or inhibit inflammatory responses.^114,115^ For vaccine development, especially for mRNA platforms, consideration of these evasion mechanisms is essential. If multi-antigen approaches can be designed, then one potential approach to generating potent and broad neutralizing antibodies, and powerful polyfunctional T-cell responses is to use both gB and PC. As demonstrated in candidates such as mRNA-1647, these antibodies and T responses could work together to overcome the MHC downregulation and NK inhibition maintained by the virus.^116^ Furthermore, such candidates could also assess immune evasion proteins, such as UL16, UL141, or vFcγRs, for their potential merit and utility in addition to or as alternative targets. Producing blocking antibodies that could restore these effector functions would also potentially prevent the virus from modulating the host immune response effectively, potentially providing enhanced protection against congenital CMV transmission, and disease among vulnerable populations.^116,117^

 The most advanced CMV vaccine candidate by Moderna is mRNA-1647. It encodes an astonishing six different mRNA transcripts—one coding for full-length gB and then five coding for the individual subunits of the PC—all co-encapsulated in the same proprietary LNP. This bundled formulation ensures that APCs and other transfected cells receive all the necessary genetic instructions to co-express and assemble both important antigenic complexes simultaneously.^118^ The outcomes of the Phase 1 and Phase 2 clinical trials of mRNA-1647 were robustly supportive of the LNP-based approach to a multivalent vaccine. The vaccine was immunogenic in both CMV-seronegative and CMV-seropositive healthy adults and generally well tolerated.^118^ A 3-dose series of the vaccine generated strong neutralizing antibody responses to infection of both fibroblasts and epithelial cells. In CMV-seronegative recipients, antibody titer levels were elevated to levels exceeding those of CMV-seropositive individuals’ levels found after natural infection, a key indicator for vaccine efficacy. The vaccine also induced strong CD4^+^ and CD8^+^T-cell responses characterized as stimulatory for long-lasting protection against herpesviruses. These favorable results supported further evaluation of mRNA-1647 in a Phase 3 efficacy trial (the “CMVictory” trial) to evaluate whether the vaccine may prevent primary CMV infection in women of childbearing age.^119^ Despite promising results of mRNA-1647, its approval by the U.S. Food and Drug Administration (FDA) will certainly require immunogenicity data, long-term safety data, and its efficacy and safety in special groups, including immunocompromised individuals and pregnant women. Regarding long-term safety, there is evidence that repeated administration leads to toxicities, such as a reduction in circulating platelet levels and an elevation in cardiac damage markers, or thymic atrophy.^120^ Additionally, specific storage and shipping conditions to ensure the efficacy and stability of mRNA vaccines need to be considered.^121^

###  mRNA vaccines for HIV

 The HIV/AIDS pandemic is still a considerable public health threat without even an efficacious prophylactic vaccine, following over 35 years of dedicated research effort. The primary challenges in developing an HIV vaccine are largely due to the biology of HIV. The HIV-1 envelope glycoprotein (Env) has a very high mutation rate, facilitating rapid epitope escape. Additionally, the Env protein has a dense “glycan shield” that protects conserved neutralizing epitopes from antibody recognition.^122^ For a vaccine to be efficacious against HIV, in other words, it must induce a rare immune response (which is difficult to achieve): the production of broadly neutralizing antibodies (bNAbs) that can recognize conserved epitopes across a variety of strains of virus, and potentially vigorous and durable T-cell responses to eliminate infected cells.^123^ HIV has developed multiple immune evasion strategies that allow for chronic infection and evasion of host immune responses, including significant genetic diversity and mutation rates in Env that allow for the HIV virus to escape neutralizing antibodies by glycan shielding or conformational masking, downregulation of MHC class I molecules by proteins, such as Nef, to limit CD8^+^T-cell recognition, avoidance of innate immune activation by mediating inhibition of type I interferon through several host factors, such as CPSF6, cyclophilins, or SAMHD1, and being able to integrate into latent compartments as a reservoir for the HIV virus within CD4^+^T-cells to go undetected.^124^ All the immune evasion strategies allow HIV to support the depletion of CD4^+^T-cells, shape DC function and allow for continued replication whilst under the pressure of immune responses.^125^ In the development of mRNA vaccines, ways to evade the immune response must be countered by developing immunogens to the use of conserved epitopes like stabilized native-like Env trimers or mosaics, capable of inducing broadly neutralizing antibodies and strong polyfunctional T-cell responses that can tolerate the viral diversity and latency, similar to what has been done in preclinical and early clinical candidates using nucleoside-modified mRNA in LNPs.^126,127^

 Preclinical studies in mice and non-human primate (NHP) systems have shown the potential of LNP-mRNA formulations for HIV. For example, an LNP-mRNA vaccine coding a stabilized Env trimer (BG505 SOSIP.664) elicited superior high titer and durable neutralizing antibody responses compared to the same protein immunized with a typical adjuvant in NHPs. These neutralizing antibodies correlated well with strong Tfh and GC B-cell responses, suggesting that LNP-delivered mRNA was a strong immune stimulant.^128^ In another study, Guenaga et al developed an mRNA-LNP platform presenting cell-surface-anchored, cleavage-independent NFL Env trimers. By linking a flexible linker (or by restoring the MPER) to the HIV gp41 transmembrane domain, the design allows for direct expression of native-like trimers on the plasma membrane. Flow cytometry verified the correct antigenic presentation of a number of different HIV Env strains following mRNA-LNP delivery *in vitro*. *In vivo*, rabbits that received four immunizations with low-dose mRNA-LNP countered with 5 µg developed strong autologous Tier-2 neutralizing antibody titers similar to those produced by matched soluble protein immunogens plus adjuvant. Epitope mapping, using electron microscopy, indicated that the vaccine-elicited antibodies predominantly targeted glycan-hole vulnerabilities and apex epitopes, and that other gp41 base responses, as seen with soluble proteins, were largely absent.^129^

 The preclinical promise has quickly transitioned into a series of landmark Phase I clinical trials, primarily led by Moderna in connection with the International AIDS Vaccine Initiative (IAVI) and the Scripps Research Institute. The IAVI G001 trial is the first-in-human trial to assess the germline-targeting approach with the eOD-GT8 60mer protein immunogen with an adjuvant. IAVI G001 trial can be considered a significant milestone since the priming immunogen successfully activated its target naive B cells in 97% of subjects.^130,131^ AVI G002 (NCT05001373) trial builds directly on G001, by administering the same germline-targeting immunogens (eOD-GT8 60mer for priming, Core-g28v2 60mer for boosting) in an mRNA-LNP platform. The primary objective is to determine whether the mRNA version will replicate the successful B-cell priming observed in G001, and possibly elicit Tfh and GC responses associated with the mRNA-LNP platform.^130^ Furthermore, the HVTN 302 (NCT05217641) trial led by Moderna assesses safety and immune responses to HIV Env immunogens delivered via mRNA-LNP technology, i.e., mRNA-1644 and mRNA-1574. mRNA-1644 encodes for the soluble Env trimers from clades B & C, while mRNA-1574 encodes the membrane-anchored Env trimers. The goal is to assess safety and immunogenicity of the immunogens, as well as to determine whether they can induce bNAbs against a variety of HIV strains.^130,132^ Due to the high variability in HIV levels and the existence of a latent reservoir, additional research and clinical trials are necessary for the development of an effective HIV mRNA vaccine that can address these issues. HIV mRNA vaccines are still in the early developmental phase and will need to undergo additional clinical testing.

###  mRNA vaccines Zika virus

 The Zika virus produces symptoms of fever, rash, malaise, pain, and conjunctivitis. Zika virus is transmitted to humans by Aedes mosquitoes, and has implications for neurodevelopmental complications and congenital birth defects because it attacks neural progenitor cells.^133^ In 2017, the researchers performed tests with an mRNA vaccine containing Zika virus membrane and envelope proteins encapsulated in LNP. They vaccinated C57BL/6 mice with a 30 µg dose. They observed a robust immune response from the vaccine and no adverse effects from immunization. The neutralizing antibody levels peaked 2 months post-vaccination, and were stable until month 3. The mRNA vaccine demonstrated effectiveness in protecting mice and primates from Zika infection in an experimental setting.^134^ Another study also assessed an mRNA vaccine with Zika membrane proteins in LNP encapsulation in a mouse model, finding that the serum had higher neutralizing antibodies, which protected the mice from Zika. Another study approached Zika in a different way by vaccinating mouse embryos on embryonic day 6 and exposing them to Zika, and found that there were lower levels of viral infection in the placenta and some tissues after two doses of an mRNA vaccine.^135,136^ While the results from animal studies of the Zika mRNA vaccines are promising, more clinical trials involving humans are necessary.

###  mRNA vaccines for rabies 

 The rabies virus is the causative agent of rabies, a viral illness in animals (and humans) that is guaranteed to result in death.^137^ The receptor for this virus is a surface protein, RABV-G, that attaches to target cells, allowing the rabies virus to invade peripheral nerves, followed by an invasion of the central nervous system. The first mRNA vaccine incorporating the RABV-G gene was tested on mice and pigs (both at the same time) in 2016. Two doses of the vaccine were administered, and neutralizing antibody levels reached at least 0.5 IU/ml. Annually, 1 month/ month, testing results showed antibody levels averaged around 40 IU/ml, which was considered strong enough protection from rabies infection.^138^ In 2017, the mRNA rabies vaccine CV7201 entered phase I of a clinical trial in younger people (18–40 years old). The results of the study stated that no serious side effects occurred in subjects receiving the vaccine; however, one subject experienced moderate Bell’s palsy. Antibody levels were based on IgM and IgG against RABV-G which showed an increasing titer with peak levels on days 21 and 42. After 12 months, IgG levels were high, indicating a strong memory immune response. CD4^+^T-cell values were above baseline during the first three months after vaccination, followed by a decline in CD4^+^T-cell values.^139^

## Conclusion

 The birth of the mRNA vaccine era, prompted by the COVID-19 pandemic, has changed the landscape of medicine and our understanding of what is possible. As we have illustrated in this review, the driving force behind this transformation is not just messenger RNA but the encapsulating delivery systems that protect it. We are now beyond believing that the carriers are just protective shells and instead understand them to be instrumental to vaccine efficacy, which is exemplified by the clinical performance of the LNP-based vaccines for SARS-CoV-2, RSV, and the imminent opportunities for influenza and CMV. One dominant platform in the current ecosystem will remain the clinically validated LNP platform, which utilizes pH-responsive ionizable lipids and has been undeniably successful in performing the critical step of endosomal escape. However, as we take aim at a more difficult target like HIV, we are now facing the limitations of current LNPs, including their liver tropism, cold-chain requirement, and potential for reactogenicity, have led to a new wave of innovation in the field. Platforms that use tunable polymeric nanoparticles allow for sustained release of antigens to support the prolonged germinal center reactions needed for bNAb development. Other approaches, including peptide-based systems and biomimetic EVs, show promise for enhanced specificity and lower immunogenicity, offering new potential therapeutic windows.

 Moving forward, the future of mRNA vaccinology will be determined by enhanced understanding and resourcefulness in the bidirectional dialogue between the delivery vehicle and the immune system. Key unmet needs and future perspectives will include: 1) Rational Design for Immune Steering: The next generation of delivery systems will be designed not for delivery success alone, but for delivery and the immunological results we seek. This will involve the design of lipid or polymer chemistry to preferentially activate a specific innate immune pathway (for example, STING vs. TLRs), to preferentially target specific classes of antigen-presenting toll-like receptors (for example, cDC1s to obtain a robust CD8^+^T-cell response), and to control antigen expression kinetics to drive B-cell affinity maturation. 2) Overcoming Logistical Barriers for Global Equity: The development of thermostable formulations using lyophilization or new carrier materials (e.g., polymers, hybrid particles) will remain the highest priority for global health. Achieving this goal will provide equitable access to mRNA technology by eliminating ultra-cold chain bottlenecks, allowing for deployment in low-resource settings. 3) Finding New Routes of Administration: Move away from intramuscular injection towards delivery through mucosal routes (e.g., intranasally), which is essential for pathogens entering humans through respiratory or genital tracts (e.g., influenza, RSV, and HIV). Finding new carriers (e.g., mucoadhesive chitosan-lipid nanoparticles) that can induce IgA at mucosal surfaces is a new frontier. 4) Navigating the Regulatory and Manufacturing Landscape: As delivery vehicles undergo complex transformations moving from simple and narrow delivery vehicles to complex and numerous options, straightforward and consistent regulatory pathways for their characterization, quality control, and safety will be essential. In parallel, a significant (but ultimately required) challenge will involve establishing scalable, cost-efficient, and reproducible, GMP manufacturing for new delivery platforms or routes such as exosomes, EVs, polyplexes, *etc*.

 In summary, the experience of the mRNA vaccine is tied to the evolution of its delivery vehicle(s), with previous success with LNPs providing an incredible starting point. The next stage will be completed with interdisciplinary scientists, which should include immunologists, material scientists, and bioengineers, simply creating smart or instructive delivery vehicles capable of tuning the immune system to overcome mankind’s last and most persistent infections.

## Competing Interests

 The authors declare no conflict of interest.

## Data availability Statement

 No data was used for the research described in the article.

## Ethical Approval

 Not applicable.
